# Marinobufagenin Inhibits Neutrophil Migration and Proinflammatory Cytokines

**DOI:** 10.1155/2019/1094520

**Published:** 2019-05-20

**Authors:** Deyse C. M. Carvalho, Luiz Henrique Agra Cavalcante-Silva, Éssia de A. Lima, José G. F. M. Galvão, Anne K. de A. Alves, Priscilla R. O. Feijó, Luís E. M. Quintas, Sandra Rodrigues-Mascarenhas

**Affiliations:** ^1^Laboratory of Immunobiotechnology, Biotechnology Center, Federal University of Paraíba (UFPB), João Pessoa, PB 58051-900, Brazil; ^2^Laboratory of Biochemical and Molecular Pharmacology, Institute of Biomedical Sciences, Health Sciences Center, Federal University of Rio de Janeiro (UFRJ), Rio de Janeiro, RJ 21941-902, Brazil

## Abstract

Cardiotonic steroids, such as ouabain and digoxin, are known to bind to Na^+^/K^+^-ATPase and to promote several biological activities, including anti-inflammatory activity. However, there are still no reports in the literature about inflammation and marinobufagenin, a cardiotonic steroid from the bufadienolide family endogenously found in mammals. Therefore, the aim of this work was to analyze, *in vivo* and *in vitro*, the role of marinobufagenin in acute inflammation. *Swiss* mice were treated with 0.56 mg/kg of marinobufagenin intraperitoneally (i.p.) and zymosan (2 mg/mL, i.p.) was used to induce peritoneal inflammation. Peritoneal fluid was collected and used for counting cells by optical microscopy and proinflammatory cytokine quantification (IL-1*β*, IL-6, and TNF-*α*) by immunoenzymatic assay (ELISA). Zymosan stimulation, as expected, induced increased cell migration and proinflammatory cytokine levels in the peritoneum. Marinobufagenin treatment reduced polymorphonuclear cell migration and IL-1*β* and IL-6 levels in the peritoneal cavity, without interfering in TNF-*α* levels. In addition, the effect of marinobufagenin was evaluated using peritoneal macrophages stimulated by zymosan (0.2 mg/mL) *in vitro*. Marinobufagenin treatment at different concentrations (10, 100, 1000, and 10000 nM) showed no cytotoxic effect on peritoneal macrophages. Interestingly, the lowest concentration, which did not inhibit Na^+^/K^+^-ATPase activity, attenuated proinflammatory cytokines IL-1*β*, IL-6, and TNF-*α* levels. To investigate the putative mechanism of action of marinobufagenin, the expression of surface molecules (TLR2 and CD69) and P-p38 MAPK were also evaluated, but no significant effect was observed. Thus, our results suggest that marinobufagenin has an anti-inflammatory role *in vivo* and *in vitro* and reveals a novel possible endogenous function of this steroid in mammals.

## 1. Introduction

Cardiotonic steroids are natural compounds capable of inhibiting Na^+^/K^+^-ATPase activity, inducing positive cardiac inotropism [1]. Marinobufagenin is a cardiotonic steroid originally isolated from *Bufo marinus* toad venom, currently called *Rhinella marina* [2]. More recently, this substance has been identified in mammalian plasma and urine, being synthesized by the adrenal gland [3, 4]. The putative physiological role of marinobufagenin as natriuretic and vasoconstrictor has also been experimentally supported [5, 6]. Consequently, this steroid is now considered a mammalian hormone [7, 8], but its endogenous functions are far from being fully understood.

Some studies suggest that cardiotonic steroids, such as ouabain, digoxin, and bufalin, have immunomodulatory activity because they interfere in several inflammatory parameters such as cell migration, vascular permeability, and proinflammatory cytokines [9–14]. The immune system, among several other functions, plays a fundamental role in the recognition and elimination of pathogenic microorganisms by different processes, as inflammation induction [15]. Inflammation is an immune response that can be triggered by pathogen- and damage-associated molecular patterns, and this process is mainly characterized by vasodilation, plasma exudation, and cell migration to the injury site, resulting in five cardinal signs: pain, redness, swelling, heat, and/or loss of function [16, 17]. Therefore, inflammation is considered as a beneficial phenomenon capable of establishing organism homeostasis. However, uncontrolled inflammation can lead to homeostatic imbalance and may evolve to chronic debilitating diseases [18].

Our group has demonstrated the ability of ouabain to act as an anti-inflammatory substance. Ouabain can reduce cell migration to the inflamed site by several stimuli, such as zymosan [19], concanavalin A [20], and pathogens like *Leishmania amazonensis* [21]. Besides, this steroid inhibits proinflammatory cytokines, such as interleukin 1*β* (IL-1*β*) and tumor necrosis factor *α* (TNF-*α*), which could be associated with p38 mitogen-activated protein kinase (MAPK p38) and nuclear factor kappa B- (NF-*κ*B-) reduced activity [22, 23]. Recently, another study of our group has shown that ouabain negatively modulates pulmonary inflammation, reducing eosinophil migration, Th2 profile cytokines, and mucus production in bronchioles [24]. Furthermore, by similar mechanisms, other studies demonstrated the anti-inflammatory effects of digoxin and bufalin [11, 12].

Although many works have investigated marinobufagenin's role in cardiovascular homeostasis, there are still no reports about marinobufagenin's effect on inflammation. Therefore, this work is aimed at analyzing marinobufagenin's effect in the acute inflammation process *in vivo* and *in vitro*.

## 2. Materials and Methods

### 2.1. Animals

Female *Swiss* albino mice (6-8 weeks) were obtained from the Thomas George animal house of Federal University of Paraíba (UFPB). Animals were kept under standard laboratory conditions on a constant 12 h light/dark cycle with temperature (21 ± 1°C), and food and water were given *ad libitum*. All procedures adopted in this study were approved by the Ethics Committee on Animal Use of UFPB (Protocol: 125/2016).

### 2.2. Isolation and Purification of Marinobufagenin

Marinobufagenin was purified from the *Rhinella schneideri* toad venom at the Institute of Biomedical Sciences (ICB) of the Federal University of Rio de Janeiro (UFRJ) as reported elsewhere [25]. A marinobufagenin sample was solubilized in dimethyl sulfoxide (DMSO) at a concentration of 5 mg/mL and stored at -20°C. For *in vivo* and *in vitro* experiments, DMSO maximum concentrations used were 0.6% and 0.016%, respectively. Ouabain was purchased from Sigma and diluted in water.

### 2.3. Marinobufagenin Pretreatment In Vivo

To compare ouabain's effect described by our group and marinobufagenin's effect, the same dose (0.56 mg/kg) and experimental protocol (pretreatment for three consecutive days) were used [19–24]. Mice were randomly divided into five experimental groups (*n* = 5): saline, DMSO, marinobufagenin (MBG), zymosan (ZYM), and MBG + ZYM. MBG and MBG + ZYM groups were treated with marinobufagenin (0.56 mg/kg) intraperitoneally (i.p.) for three consecutive days. All groups received 0.5 mL of each solution. MBG dose and administration route were chosen based on the previous work from our group, wherein a dose-response curve was performed using 0.10, 0.31, and 0.56 mg/kg of ouabain, another physiological cardiotonic steroid, for three consecutive days in a model of ZYM-induced paw edema [19].

### 2.4. Zymosan-Induced Peritonitis

Peritoneal inflammation was performed as described in the literature [26, 27]. Zymosan A was freshly prepared (2 mg/mL) in sterile saline, and 0.5 mL was injected i.p. one hour after the last injection on day 3. Four hours after zymosan stimulus, animals were euthanized by cervical displacement. The peritoneal cavity was then lavaged with 3 mL of cold phosphate-buffered saline (PBS), and exudates were collected.

### 2.5. Total and Differential Cell Count

Exudates collected were pooled, and the total number of leukocytes was determined by optical microscopy using Turk's solution (0.01% crystal violet in 3% acetic acid). For differential cell count, samples were centrifuged at 350*g* for 10 minutes (4°C). Cytospin slides from each sample were stained by Fast Panoptic Method. Differential cell count was performed by optical microscopy, and each slide was analyzed until the count of 100 cells can be reached using an oil-immersion objective. Finally, the supernatant was collected and stored at −20°C for cytokine analysis.

### 2.6. Measurement of Cytokine Levels by Enzyme-Linked Immunosorbent Assay (ELISA)

IL-1*β*, IL-6, and TNF-*α* cytokines present in peritoneal fluid were quantified by sandwich ELISA according to the manufacturer's instructions (eBioscience). Optical density was read at 450 nm using a microplate spectrophotometer (microplate reader VersaMax, tunable, BN2529, Molecular Devices).

### 2.7. In Vitro Tests

Peritoneal macrophages of *Swiss* albino mice were used for *in vitro* experiments. Thus, peritoneal exudate was elicited in mice with an i.p. injection of 4% thioglycolate (Sigma-Aldrich). Four days after thioglycolate injection, animals were euthanized by cervical displacement and peritoneal cavity was washed with 5 mL of complete RPMI-1640 medium (Gibco) (streptomycin: 10 mg/mL, penicillin: 6 mg/mL, and kanamycin: 2 mg/mL), supplemented with 10% fetal bovine serum (FBS) (Gibco). Cell suspension obtained from peritoneal lavage was centrifuged at 350*g* for 5 minutes (4°C). The supernatant was discarded, and the pellet was resuspended in 2 mL of complete RPMI medium. Viable cells were counted with a Neubauer chamber (Hemocytometer L. Optik ATC-111020) using a Trypan Blue solution (Merck). Macrophages were then enriched by adherence to plastic. For that, viable peritoneal cells were seeded in 96-well plates at a concentration of 4 × 10^5^ cells/well in a final volume of 200 *μ*L and incubated overnight (18 h) in an atmosphere of 5% CO_2_ at 37°C. Then, nonadherent cells were removed by aspiration. Remaining cells were stimulated with zymosan (0.2 mg/mL) and treated with different marinobufagenin concentrations (10, 100, 1000, and 10000 nM), the same used in ouabain experiments [19]. After 24 h of culture, macrophages were used for cell viability test, and the supernatant was removed for the quantification of cytokine levels (IL-1*β*, IL-6, and TNF-*α*) by ELISA, as previously described.

### 2.8. Macrophage Viability Analysis

Cell viability was estimated by MTT (3-[4,5-dimethylthiazol-2-yl]-2,5-diphenyltetrazolium bromide) assay. For that, 100 *μ*L of RPMI medium containing 10% of MTT solution (5 mg/mL) was added to each well and cells were maintained in an atmosphere of 5% CO_2_ at 37°C. After 4 h of incubation, the MTT-containing medium was removed, and the precipitate was solubilized in the DMSO solution (100 *μ*L). Optical density was read at 570 nm using a microplate spectrophotometer (microplate reader VersaMax, tunable, BN2529, Molecular Devices).

### 2.9. TLR2 and CD69 Expression Evaluation

Peritoneal macrophages isolated as described in Section 2.7 were placed in 6-well plates (1.5 × 10^6^/well) and treated with marinobufagenin (10 nM) in the absence or presence of zymosan (0.2 mg/mL) for 24 h. After this period, cells were collected and placed in a 96-well plate (U bottom). Then, cells were blocked to prevent nonspecific binding using anti-CD16/32 and labeled separately with antibodies anti-TLR2 (PE) and anti-CD69 (PE), according to the manufacturer's instructions (BD Biosciences). Finally, cells were suspended in PBS and evaluated in a flow cytometer—FACSCanto II (Becton & Dickinson).

### 2.10. P-p38 MAPK Evaluation

The same culture conditions were performed for P-p38 MAPK evaluation, as described in Section 2.9. After a 24 h period in culture, cells were placed in a 96-well plate (U bottom). Then, cells were fixed by Cytofix (BD Cytofix™) and permeabilized by Perm Buffer (BD Phosflow™) for 30 min each. Subsequently, cells were labeled with antibody anti-P-p38 (PE-Cy7), according to the manufacturer's instructions (BD Biosciences). Finally, cells were suspended in PBS and evaluated in a flow cytometer—FACSCanto II (Becton & Dickinson).

### 2.11. Flow Cytometry Data Analysis

Briefly, peritoneal macrophages were visualized using size (FSC), and granularity (SSC) parameters and a gate were performed in the cell population. Macrophages were analyzed by fluorescence intensity quantification corresponding to antibody labeling. These data were evaluated using FlowJo software version 10.

### 2.12. Determination of Na^+^/K^+^-ATPase Activity

Crude homogenates of naive mouse brain (source of cardiotonic steroid-sensitive Na^+^/K^+^-ATPase *α*2 and *α*3 isoforms, in rodents) and kidneys (source of cardiotonic steroid-resistant Na^+^/K^+^-ATPase *α*1 isoform, in rodents) were performed as described by Müller et al. [28]. Briefly, the brain and kidneys were homogenized in a buffer containing sucrose 250 mM, EGTA 10 mM, EDTA 1 mM, dithiothreitol 0.5 mM, PMSF 0.1 mM, and Tris–HCl 50 mM (pH 7.4) using a Potter apparatus. Homogenates were ultracentrifuged at 100000*g* for 60 min in an Optima XE-90 ultracentrifuge (Beckman Coulter). The pellet was resuspended in the same buffer without PMSF and stored at -80°C. Protein concentration was determined by the Lowry protein assay method. According to Noël et al. [29], with slight modifications, in a buffered medium containing 97.6 mM NaCl, 3 mM KCl, 3 mM MgCl_2_, 3 mM ATPNa_2_, 1 mM EGTA, and 20 mM maleic acid/Tris (pH 7.4), brain and kidney preparations were incubated at 37°C with increasing concentrations of marinobufagenin or ouabain. After 2 h, the reaction was stopped by the Fiske & Subbarow solution to measure the Pi released from ATP hydrolysis by a colorimetric method.

### 2.13. Statistical Analysis

Inhibition curves were fitted by nonlinear regression analysis (GraphPad Prism 7.0; GraphPad Software Inc.) assuming a sigmoidal concentration-response curve model of one site (kidney/ouabain and brain/marinobufagenin) or two sites (brain/ouabain), and the mean inhibitory concentration (IC_50_) was estimated. Data were expressed as mean ± SEM and analyzed by GraphPad Prism 7.0 software. Results were submitted to one-way analysis of variance (ANOVA) followed by Tukey's test. Results were considered statistically significant when *p* < 0.05.

## 3. Results

### 3.1. Marinobufagenin Reduced Cell Migration to the Peritoneal Cavity

**Figure 1:**
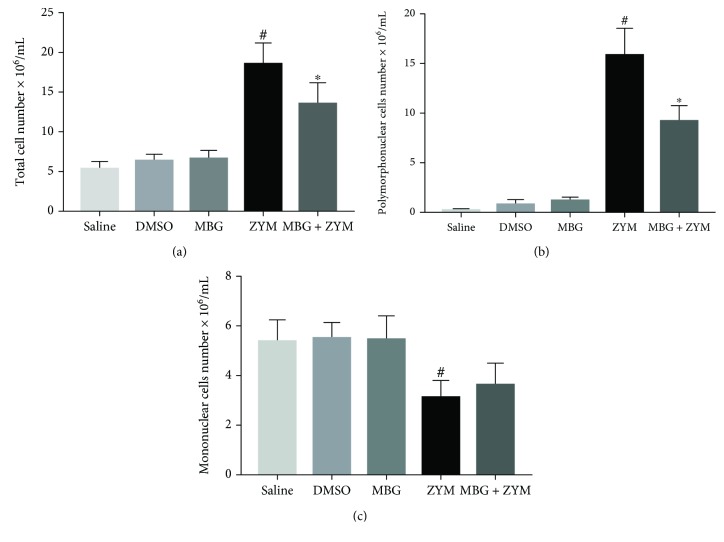
Marinobufagenin's effect on leukocyte migration to the peritoneal cavity. *Swiss* mice were pretreated for three consecutive days with marinobufagenin (0.56 mg/kg), saline, or DMSO. One hour after the last day of pretreatment, animals were stimulated with zymosan at 2 mg/mL concentration. Four hours after of zymosan stimulus, peritoneal exudate was collected and total and differential leukocyte numbers were evaluated. (a) Total cell number. (b) Polymorphonuclear cell number. (c) Mononuclear cell number. Numerical data were presented as mean ± e.p.m of two experiments with *n* = 5 and analyzed by one-way analysis of variance (ANOVA) followed by Tukey posttest. ^#^*p* < 0.05, significant when compared to the saline group. ^∗^*p* < 0.05, significant when compared to the zymosan group. DMSO: dimethyl sulfoxide; MBG: marinobufagenin; ZYM: zymosan.

As expected, zymosan stimulus led to an increase in total cell number in the peritoneal cavity compared to the saline group. On the other hand, marinobufagenin pretreatment decreased zymosan-induced leukocyte migration to the peritoneum by 27% (Figure 1(a)). This effect was related to inhibition of polymorphonuclear leukocyte migration by marinobufagenin when compared to the zymosan group (42%, Figure 1(b)). Additionally, marinobufagenin pretreatment did not interfere with the number of mononuclear leukocytes when compared to the zymosan group (Figure 1(c)). In DMSO and marinobufagenin groups (unstimulated animals), cell number remained similar to the saline group (Figures 1(a)–1(c)).

### 3.2. Marinobufagenin Modulated Proinflammatory Cytokines In Vivo

**Figure 2:**
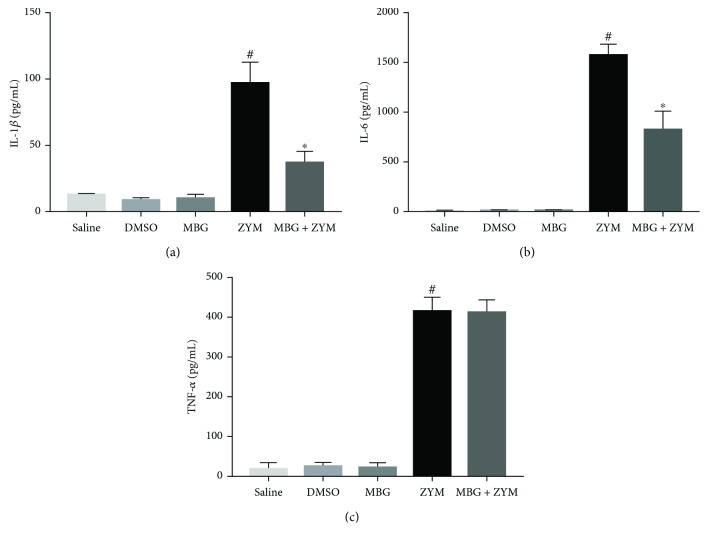
Marinobufagenin's effect on proinflammatory cytokines *in vivo*. *Swiss* mice were pretreated for three consecutive days with marinobufagenin (0.56 mg/kg), saline, or DMSO. One hour after the last day of pretreatment, animals were stimulated with zymosan at 2 mg/mL concentration. Four hours after zymosan stimulus, peritoneal exudate was collected and centrifuged. Cytokine levels were quantified from exudate supernatant by enzyme immunoassay (ELISA). (a) IL-1*β*, (b) IL-6, and (c) TNF-*α*. Numerical data were presented as mean ± e.p.m of two experiments with *n* = 3 and analyzed by one-way analysis of variance (ANOVA) followed by Tukey posttest. ^#^*p* < 0.05, significant in relation to the saline group. ^∗^*p* < 0.05, significant in relation to the zymosan group. DMSO: dimethyl sulfoxide; MBG: marinobufagenin; ZYM: zymosan.

According to previous data, zymosan stimulus induces an increase in proinflammatory cytokines in the peritonitis model [30]. Indeed, as shown in Figure 2, zymosan-stimulated animals showed a significant increase in three proinflammatory cytokines (IL-1*β*, IL-6, and TNF-*α*) compared to the saline group. Marinobufagenin pretreatment in zymosan-challenged animals induced a reduction in IL-1*β* (62%) and IL-6 (48%) but not TNF-*α* levels when compared to the zymosan group. In DMSO and marinobufagenin groups (unstimulated animals), cytokine levels remained similar to the saline group (Figures 2(a)–2(c)).

### 3.3. Marinobufagenin Cytotoxicity in Peritoneal Macrophages

**Figure 3:**
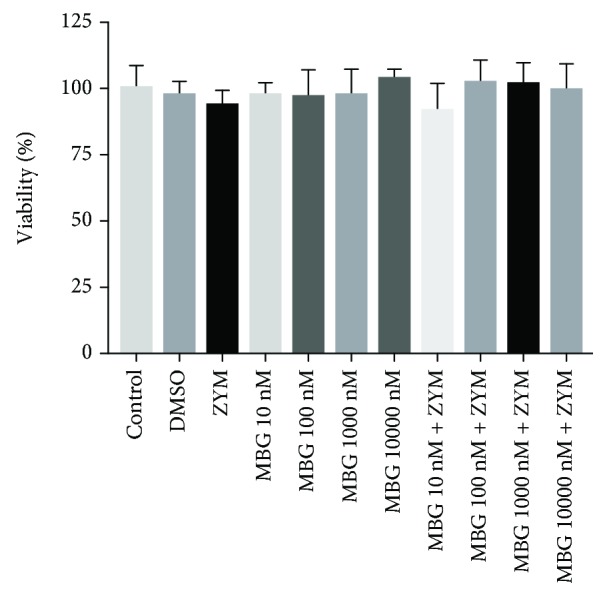
Marinobufagenin's effect on macrophage viability. Peritoneal macrophages were stimulated with zymosan (0.2 mg/mL) and treated with different marinobufagenin concentrations (10, 100, 1000, and 10000 nM). After 24 h of culture, cells were used for cell viability test by MTT assay. Numerical data were presented as mean ± e.p.m of two experiments with *n* = 3 and analyzed by one-way analysis of variance (ANOVA) followed by Tukey posttest. DMSO: dimethyl sulfoxide; ZYM: zym; MBG: marinobufagenin.

Cultured peritoneal macrophages were used to evaluate marinobufagenin cytotoxicity. As shown in Figure 3, cell viability was not affected by 10 to 10000 nM marinobufagenin in absence or presence of zymosan when compared to the control group (culture medium).

### 3.4. Inhibitory Profile of Marinobufagenin on Mouse Na^+^/K^+^-ATPase *α*-Isoforms

**Figure 4:**
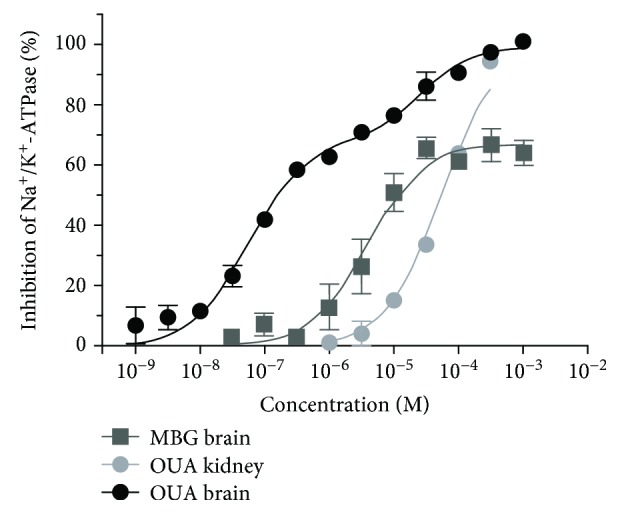
Inhibition curves of Na^+^/K^+^-ATPase from the mouse brain and kidney preparations by marinobufagenin and ouabain. Crude membrane preparations were incubated for 2 h at 37°C with increasing concentrations of marinobufagenin or ouabain, and Pi was analyzed by a colorimetric method. Results were expressed as percent of the inhibition measured in the presence of 1 mM ouabain (mean ± SEM) and were obtained from at least 2 independent experiments performed in triplicate. Curves were built by nonlinear regression analysis. MBG: marinobufagenin; OUA: ouabain.

Inhibition curves representing the effect of increasing concentrations of marinobufagenin (and ouabain, for comparison) on mouse Na^+^/K^+^-ATPase *α*1 (kidney preparation) and mostly *α*2 and *α*3 (brain preparation) isoforms are depicted in Figure 4. As we can see, the ouabain curve for the mouse brain has two components, a major one (around 70% of inhibition) composed by *α*2/*α*3-sensitive isoforms (IC_50_ = 55 ± 9 nM) and a minor one which reflects the *α*1-resistant isoform (IC_50_ = 25 ± 12 *μ*M). The latter was similar to the potency estimated for the mouse kidney, known to harbor only the *α*1-resistant isoform (IC_50_ = 53 ± 5 *μ*M). In contrast, just a single sigmoidal curve was observed for the mouse brain when marinobufagenin was employed, reaching a maximal inhibitory effect at 70% (IC_50_ = 3.8 ± 0.9 *μ*M), which reflects a 70-fold lower potency of marinobufagenin to inhibit *α*2/*α*3 isoforms and failure to inhibit *α*1 (Figure 4). Consistently, the mouse kidney *α*1 isoform could not be inhibited at the highest concentration of marinobufagenin (1 mM).

### 3.5. Marinobufagenin Modulated Proinflammatory Cytokines In Vitro

**Figure 5:**
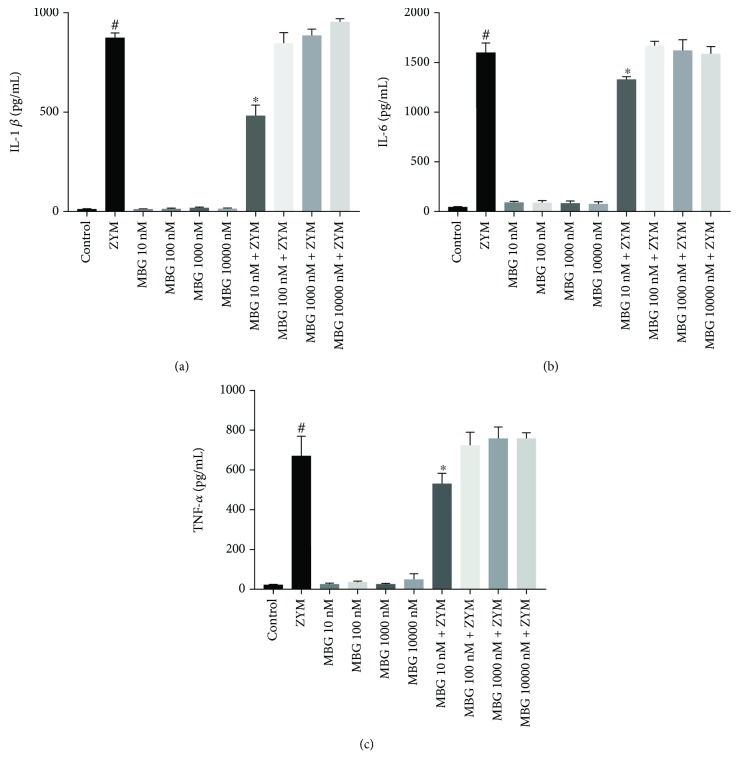
Marinobufagenin's effect on proinflammatory cytokines *in vitro*. Peritoneal macrophages were stimulated with zymosan (0.2 mg/mL) and treated with different marinobufagenin concentrations (10, 100, 1000, and 10000 nM). After 24 h of culture, the supernatant was removed for cytokine level quantification (IL-1*β*, IL-6, and TNF-*α*) by ELISA. (a) IL-1*β*, (b) IL-6, and (c) TNF-*α*. Numerical data were presented as mean ± e.p.m of two experiments with *n* = 3 and analyzed by one-way analysis of variance (ANOVA) followed by Tukey posttest. ^#^*p* < 0.05, significant in relation to the saline group. ^∗^*p* < 0.05, significant in relation to the zymosan group. ZYM: zymosan; MBG: marinobufagenin.

In addition to *in vivo* analysis, the effect of marinobufagenin on proinflammatory cytokines was also evaluated *in vitro*. Zymosan-stimulated peritoneal macrophages showed increased proinflammatory cytokine levels (IL-6, IL-1*β*, and TNF-*α*) compared to the control group. Marinobufagenin treatment at the lowest concentration (10 nM) decreased IL-1*β* (45%), IL-6 (17%), and TNF-*α* (20%) levels when compared to the zymosan group. However, other concentrations did not alter the level of these cytokines. In the DMSO (data not shown) and marinobufagenin groups (unstimulated cells), cytokine levels remained similar to the control group (Figures 5(a)–5(c)).

### 3.6. Marinobufagenin's Effect on TLR2 and CD69 Expression and p38 MAPK Phosphorylation

**Figure 6:**
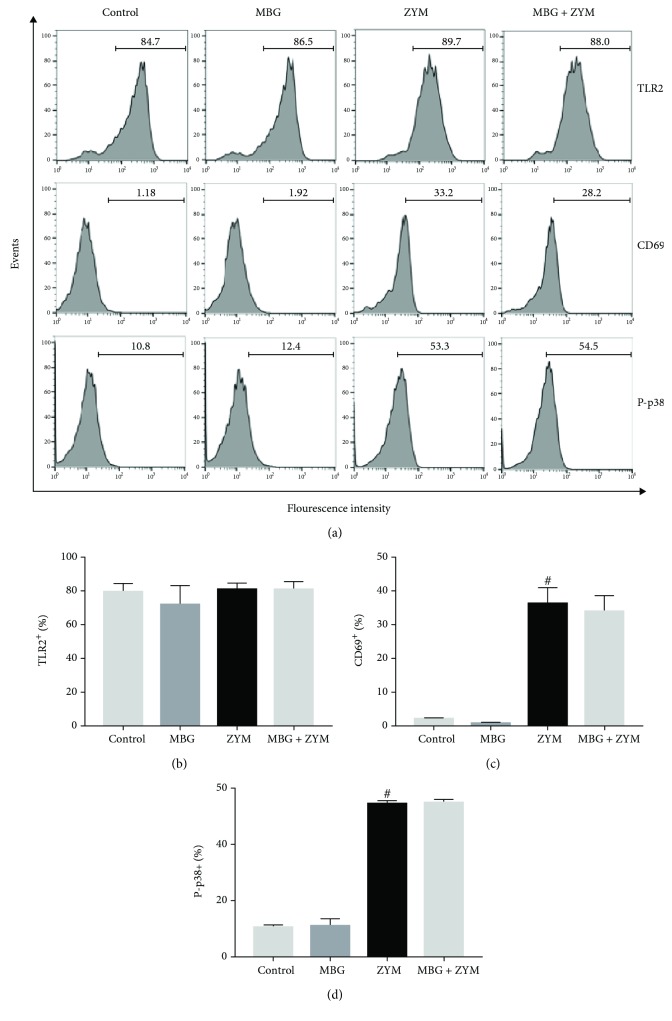
Marinobufagenin's effect on surface molecules' expression and p38 phosphorylation. Peritoneal macrophages were stimulated with zymosan (0.2 mg/mL) and treated with marinobufagenin concentrations 10 nM for 24 h. Cells were labeled separately with antibodies anti-TLR2, anti-CD69, and anti-P-p38 and analyzed by a flow cytometer. (a) Representative fluorescence histograms, (b) TLR2^+^ (%), (c) CD69^+^ (%), and (d) P-p38^+^ (%). Numerical data were presented as mean ± e.p.m of two experiments with *n* = 3 and analyzed by one-way analysis of variance (ANOVA) followed by Tukey posttest. ^#^*p* < 0.05, significant in relation to the control group. ZYM: zymosan; MBG: marinobufagenin.

To investigate the mechanism of action of marinobufagenin, TLR2, CD69, and P-p38 MAPK expression analyses were performed. Zymosan stimulus significantly increased CD69 expression in peritoneal macrophages. On the other hand, it did not interfere in TLR2 expression, as compared to the control group. Marinobufagenin treatment did not modulate the expression of these two cell surface molecules (TLR2 and CD69) when compared to the zymosan group (Figures 6(a)–6(c)). Also, zymosan induced p38 phosphorylation in peritoneal macrophages and marinobufagenin did not prevent p38 activation (Figures 6(a) and 6(d)).

## 4. Discussion

Several works have demonstrated that cardiotonic steroids have a role in the immune system acting as an anti-inflammatory substance [9–13]. Marinobufagenin, a mammalian hormone described as a blood pressure regulator, has not been tested in inflammation so far. Here, we evaluated for the first time the effect of marinobufagenin in the acute inflammatory process using the zymosan-induced peritonitis model (*in vivo*) and cultured peritoneal macrophages (*in vitro*).

Zymosan-induced peritonitis represents an acute inflammation model characterized by increased vascular permeability and production of inflammatory mediators, leading to migration of leukocytes, mainly polymorphonuclear cells (neutrophils) in the peritoneum for the first four hours [27]. In accordance with previous data, we observed that zymosan injection induced neutrophil migration to the peritoneal cavity, and marinobufagenin pretreatment was able to reduce it significantly. These data are similar to several studies performed by our group using ouabain which decreased polymorphonuclear cell migration in peritoneal inflammation induced by zymosan [19], concanavalin A [20], and *Leishmania amazonensis* [21] and also in the pulmonary inflammation model induced by ovalbumin (OVA) [24]. This ability to inhibit cell migration may also be related to vascular permeability, since it was seen that this cardiotonic steroid has a vasoconstricting action in pulmonary and mesenteric arteries [5]. Besides, a study with CTB (cytotrophoblast) and CHO (Chinese hamster ovary) cells demonstrated that low concentrations of marinobufagenin (10 and 100 nM) impaired cell migration in transwell assay [31].

Peritoneal macrophages (mononuclear cells) are resident in the peritoneum, and their activation leads to the release of several mediators and results in increased vascular permeability and leukocyte extravasation [32]. Zymosan injection causes an initial decline in peritoneal macrophage number. This phenomenon is described as a macrophage disappearance reaction and is possibly due to activation and consequently enhanced adherence of these cells in the inner layers of the peritoneum [33–35]. This event was observed here in which zymosan-stimulated animals presented less mononuclear cells in the peritoneal exudate. Marinobufagenin pretreatment in stimulated animals was not able to interfere in this process and, thus, resident macrophage activation. These data are similar to our previous findings with ouabain [19].

It has been well described that leukocyte recruitment and inflammation maintenance involve proinflammatory cytokines such as IL-1*β*, IL-6, and TNF-*α* in zymosan-induced peritonitis [30]. Our data demonstrated that zymosan injection stimulated the production of these three proinflammatory cytokines. Marinobufagenin pretreatment reduced IL-1*β* and IL-6 levels present in peritoneal exudate without changing TNF-*α* levels. In this same model, Leite et al. [19] described that ouabain blunted IL-1*β* and TNF-*α* but not IL-6 levels. Differences of the affected cytokines may be associated with the pharmacological profile of these steroids. We have recently shown that structurally related bufadienolides generate distinct cellular effects probably by a particular selection of intracellular signaling pathways, which is called functional selectivity [25]. At last, the reduction in cytokine levels and polymorphonuclear migration caused by marinobufagenin suggests an anti-inflammatory role *in vivo.*

Incubation of cultured peritoneal macrophages with 10 nM-10 *μ*M marinobufagenin for 24 h displayed no cytotoxicity. This is compatible with our data using ouabain, which did not alter macrophage viability at least up to 100 nM [19]. Micromolar concentrations of cardiotonic steroids are capable of inducing cell death through Na^+^/K^+^-ATPase inhibition [36, 37]. Rodents like mouse and rat are almost 1000 times less sensitive to these steroids when compared to other mammals because of differences in the amino acid sequence of the H1-H2 extracellular domain of the most abundant, housekeeping Na^+^/K^+^-ATPase *α*1 isoform [38]. This isoform is possibly the only one expressed in peritoneal macrophages [39]. Additionally, we have shown here and elsewhere [25, 40] that marinobufagenin is 20-80 times less potent than ouabain. Indeed, 1 mM marinobufagenin did not inhibit the murine Na^+^/K^+^-ATPase *α*1 isoform, and the inhibition of the other isoforms, *α*2 and *α*3, only begins at around 1 *μ*M (IC_50_ = 3.8 *μ*M). Marinobufagenin has been considered a unique cardiotonic steroid regarding its selectivity to the ouabain-resistant rodent *α*1 isoform; namely, in contrast to any other bufadienolide or cardenolide, it was shown to be more potent to inhibit the rat Na^+^/K^+^-ATPase *α*1 isoform than *α*2/*α*3 isoforms [41], and it has been claimed as an endogenous Na^+^/K^+^-ATPase *α*1 isoform ligand [42]. Nevertheless, we [25, 43] and others [44] have found that marinobufagenin has a selectivity profile against rodent Na^+^/K^+^-ATPase *α* isoforms similar to all known cardiotonic steroids (*α*2/*α*3 > *α*1) and is a poor *α*1 ligand. The reason of these discrepancies is unknown and is now being better investigated. Interestingly, the lowest marinobufagenin concentration (10 nM) was the single one that partly hampered zymosan-induced peritoneal macrophage IL-1*β*, IL-6, and TNF-*α* increased levels *in vitro*. Besides Na^+^/K^+^-ATPase inhibition, cardiotonic steroids may modulate intracellular signaling cascades by protein-protein interaction mediated by Na^+^/K^+^-ATPase but independent on changes in ionic gradients [45]. Namely, concentrations that do not inhibit the enzyme may activate intracellular messengers. Immune system cells recognize zymosan via Toll-like receptor 2 (TLR2). This recognition induces an intracellular signaling cascade involving MAPK phosphorylation, such as p38 [46, 47]. MAPK activation positively regulates transcription factors, which bind to DNA and induce increased expression of costimulatory molecules on the cell surface, such as CD69 [48], and promotes cytokine regulation such as TNF-*α*, IL-1*β*, and IL-6. In addition to its role as a cell activation marker, CD69 is also involved in inflammatory signaling pathways that result in the release of inflammatory mediators, such as cytokines [49, 50]. Our results showed that zymosan stimulation of peritoneal macrophages was, in fact, capable of upregulating the CD69 surface molecule and evoking the phosphorylation (activation) of p38. Nevertheless, marinobufagenin did not affect TLR2 and CD69 expression or p38 activation. Unlike marinobufagenin, our group, using murine thymocytes and monocytes, showed that ouabain was able to enhance CD69 expression and reduce p38 phosphorylation [22, 51]. These different effects found in the intracellular signaling pathway may be related to the structural differences between these compounds. Marinobufagenin has fewer hydroxyl groups when compared to ouabain. In addition, it does not have a sugar group on carbon 3 (C3) and presents an epoxide grouping between C14 and C15 in its structure [25]. Furthermore, it was demonstrated that binding of ouabain and marinobufagenin to Na^+^/K^+^-ATPase leads to different conformational changes in this enzyme [52]. Thus, it is possible that distinct conformational changes in Na^+^/K^+^-ATPase may trigger different cellular signaling pathways by functional selectivity [25]. Therefore, it is suggested that the marinobufagenin-induced pathway in peritoneal macrophages is independent on p38 signaling but might be related to another MAPK or directly to transcription factors. In this context, marinobufagenin's ability to attenuate cytokine levels in peritoneal macrophage culture supports the role of this substance as an anti-inflammatory agent *in vitro*.

## 5. Conclusion

In conclusion, our findings demonstrate the role of marinobufagenin in the immune system, evidencing its anti-inflammatory effect *in vivo* and *in vitro*. This study contributes to clarifying the physiological functions of cardiotonic steroids. Finally, more studies are needed to better understand the mechanism of action of marinobufagenin.

## Data Availability

The data used to support the findings of this study are available from the corresponding author upon request.
